# Comorbidites in NPH - local introspective - ‘Shunt them all’!

**DOI:** 10.1186/2045-8118-12-S1-P18

**Published:** 2015-09-18

**Authors:** Patricia Anne Haylock-Vize, Eleanor Carter, Syed Shah, Claudia Craven, Aswin Chari, Simon Thompson, Edward W Dyson, Samir Matloob, Andrew Stevens, Huan Wee Chan, Jinendra Ekanayake, Ahmed Toma, Michelle Leemans, Laurence D Watkins

**Affiliations:** 1 The National Hospital for Neurology and Neurosurgery, UK

## Introduction

In response to the 2013 ISH-CSF task force review on comorbidities in NPH we assessed 73 patients who were diagnosed with NPH and underwent shunt surgery at our tertiary neurosurgical unit between August 2008 and August 2012.

## Method

Data was collected as a retrospective case analysis and converted to the ICD-10 Charlson Comorbidity Index (CCI) which predicts the ten-year mortality for a patient with a range of comorbid conditions and the American Society of Anaesthesiologists (ASA) grade.

Factors assessed past medical history, polypharmacy, pre-operative haemoglobin, duration and NPH symptoms, exercise tolerance, functional ability, complications, care level of admission, length of stay, destination on discharge and allied health professional input.

## Results

CCI average was 5.5 with a range of 3 - 10 (Mild CCI 1-2, moderate CCI 3-4, Severe CCi >5).

ASA average was 2.66 ranging from 2-4.

Over a 4 year period, this patient group amassed 573 hospital days with an average of 8.18 days each patient ranging from a minimum of 3 days to a maximum of 26 days.

Discharge destination; 61 (83.56%) patients went straight home from point of discharge, 6 (8%) patients were admitted to rehabilitation unit, 3 (4%) patients returned to their referring hospital, 1 patient returned to a nursing home and 1 patient returned to sheltered accommodation.

11 (15%) patients were dependent for all ADLs, 43 (59%) patients required help with ADLs and 20 (27%) patients were fully independent.

Only 2 patients did not require any allied health care professional input during their stay indicating 97% of these patient do require AHP in put.

43% have had their presenting symptoms for less than 12 months at the time of treatment. 68% had symptoms for 24 months at time of treatment.

## Conclusion

Our NPH patient cohort present with a severe CCI score necessitating adequate neuroanaesthetic pre-operative review and work up. Most neurosurgical units exclude patients for shunt based on comorbidity, yet our data indicates our group of patients to be a complex group who we do not exclude based on comorbidity where they have demonstrated a good response to simulated shunt through extended lumbar drainage protocol.

This patient group rely heavily on Allied Healthcare Professionals in correlation with their in-patient episode.

Most patients (68%) received treatment within 2 years of symptom onset demonstrating capacity to target patents in the less than 1 year symptom onset time period.

By understanding the needs of our NPH patient population it puts us in a better position to design our service to support our patient needs and facilitate an optimal hospital episode.
